# Oral Administration of a *Shigella* 2aT32-Based Vaccine Expressing UreB-HspA Fusion Antigen With and Without Parenteral rUreB-HspA Boost Confers Protection Against *Helicobacter pylori* in Mice Model

**DOI:** 10.3389/fimmu.2022.894206

**Published:** 2022-06-13

**Authors:** Xin Zhang, Shuli Sang, Qing Guan, Haoxia Tao, Yanchun Wang, Chunjie Liu

**Affiliations:** ^1^ State Key Laboratory of Pathogen and Biosecurity, Institute of Biotechnology, Academy of Military Medical Sciences, Beijing, China; ^2^ Department of Pharmacy, Medical Supplies Center, Chinese People's Liberation Army General Hospital, Beijing, China

**Keywords:** *Helicobacter pylori*, vaccine, UreB, HspA, immune protection

## Abstract

*Helicobacter pylori* (*H. pylori*) is a gram-negative pathogen classified as a class I carcinogen. The *H. pylori* urease B subunit (UreB) and heat shock protein A (HspA) are two important vaccine candidate antigens. In this study, we evaluated the immunogenicity and immunoprotective effect of the attenuated *Shigella* vector vaccine SH02 expressing the UreB-HspA fusion protein of *H. pylori* in a mouse model. Oral SH02 with or without subcutaneous injection of rUreB-HspA induced antigen-specific serum IgG, mucosal sIgA, and T cells immune response. Subcutaneous injection of the candidate antigen rUreB-HspA enhanced the level of serum antigen-specific IgG antibodies (*p* < 0.0001) and the levels of IgG1/IgG2a/IgG2b subtypes. In addition, injection boost also increased the proportion of spleen antigen-specific CD4+CD154+ T cells (*p* < 0.001), and the proportion of CD4+CD154+ T cells that secrete IFN-γ and IL-17A. Following the *H. pylori* challenge, the levels of *H. pylori* colonization in the two experimental groups (Groups A and B) significantly reduced compared with the control group (*p* < 0.001), indicating that the candidate vaccine yielded a preventive effect of anti-*H.pylori* infection. Compared with the non-subcutaneous booster injection group (Group A), the subcutaneous booster injection group (Group B) exhibited less gastric inflammation, but there was no significant difference in the level of colonization (*p* > 0.05). These results lay a foundation for the development of a vaccine against *H. pylori* and the optimization of immunization methods and procedures to prevent *H. pylori* infection.

## Introduction

*Helicobacter pylori* (*H. pylori*) is a spiral-shaped, gram-negative bacterium that colonizes the stomachs of approximately half of the world’s population, causing chronic gastritis and peptic ulcers, and potentially predisposing the development of gastric cancer and mucosa-associated lymphoid tissue lymphoma (1). *H. pylori* was categorized in 1994 as a class I carcinogen by the World Health Organization’s (WHO) International Agency for Research on Cancer (IARC) (2). Contact with infected people directly or through contaminated water is the main route of transmission, with transmission usually occurring within the family, especially in children. The prevalence of *H. pylori* in developing countries is much higher than in developed countries (3). The current treatment for *H. pylori* infection is triple or quadruple therapy including antibiotics and proton pump inhibitors (PPI), for example amoxicillin + clarithromycin + PPI or bismuth salicylate + metronidazole + tetracycline + PPI (4). However, the increase in drug resistance rates, poor medication compliance, high cost, and reinfection are still the main reasons that the infection rate cannot be reduced (5). Moreover, long-term use of antibiotics in children, the elderly, and immunocompromised persons may cause long-term disruption of the gastrointestinal microbiome (6). The development of preventive or therapeutic vaccines may be a desirable way to control *H. pylori*-induced gastric disease, but there is still no licensed vaccine on the market.

Urease is an important virulence factor necessary for *H. pylori* to survive in and colonize the stomach, which catalyzes the decomposition of urea into ammonia and carbon dioxide, neutralizing gastric acid (7). Urease is composed of UreA and UreB subunits, consisting of up to 6% *H. pylori* protein (8). UreB is one of the effective and common antigens of *H. pylori*, and has been extensively investigated as a potential antigen for the development of vaccines against *H. pylori* infection (9–11). *H. pylori* heat shock protein A (HspA) is a bacterial heat shock chaperone with essential function as a Ni-ion scavenging protein, which is another key virulence factor and protective antigen against *H. pylori* infection (12). HspA is involved in intracellular nickel sequestration and detoxification, which is an important co-factor required for the maturation and enzymatic activity of *H. pylori* urease (13).

In addition to protective antigens, antigen delivery systems are also key factors that stimulate vaccines to produce an effective immune response. The *Shigella flexneri* 2a mutant strain has been widely used as a live attenuated vaccine strain because it is safe, effective, and can be administered orally. *Shigella flexneri* 2a strain T32 was a pioneering *Shigella* vaccine strain developed by Istrati in Romania, and it was shown to be well tolerated and probably protective by oral administration (14). At the same time, the infection of attenuated *Shigella flexneri* 2a is limited to the digestive system and cannot be transmitted into the circulatory system, which is used as a vector for delivering protective antigens against pathogens (15, 16).

An effective vaccine usually requires more than one time immunization in the form of prime-boost. Traditionally the same vaccines are given multiple times as homologous boosts. In many cases, heterologous prime-boost with different types of vaccines containing the same antigens can be more immunogenic than homologous prime-boost (17, 18). By now, prime-boost strategy has been used in many vaccines development, especially live attenuated vaccines (19). In previous studies, we have constructed an attenuated *Shigella flexneri* 2aT32 vector vaccine SH02 against *H. pylori* that intracellularly expresses the UreB-HspA fusion protein antigen of *H. pylori* using the asd-based chromosome-plasmid balanced lethal system (20). In this study, we designed two different prime-boost strategies: two oral immunization as the basic immunization, followed by homologous boost or heterologous boost, evaluated the immunogenicity and the immunoprotective effect of two immunization strategies, and explored the potential immune protection mechanism.

## Materials and Methods

### Bacterial Strains and Culture Conditions

The attenuated vector vaccine SH02 against *H. pylori* was prepared by our laboratory and stored at –80°C in Luria-Bertani (LB) containing 30% glycerol. In each experiment, SH02 was recovered to room temperature from the freezing solution, 50 µL was inoculated into 5 mL antibiotic-free LB medium, and it was incubated with shaking overnight at 37°C. The following day, 2 mL of the bacterial solution was added to 200 mL of antibiotic-free LB medium, incubated with shaking at 37°C for three hours, and induced with 1 mM IPTG for four hours. The expression of the rUreB-HspA fusion protein was assessed by SDS-PAGE and Western Blot. Vector strain was expressed and identified in the same way. The bacteria of SH02 were subsequently collected by centrifugation, washed once with sterile phosphate-buffered saline (PBS), and resuspended with sterile PBS to a final concentration of about 1.25 × 10^10^ cfu/mL.

*H. pylori* strains SS1 were cultured on campylobacter agar base plates (CDRC, Shanghai, China) with 7% fetal bovine serum at 37°C under microaerophilic conditions with 5% O_2_, 10% CO_2_, and 80% N_2_. Three days later, colonies were scraped and washed twice with cold PBS and pelleted by centrifugation at 8000 × g at 4°C for 10 min. Then, the bacterial pellets were resuspended in sterile PBS, the OD value was measured, and the concentration was estimated and adjusted to an appropriate concentration to inoculate mice.

### Construction, Expression, and Purification of *H. pylori* Recombinant Antigens

The gene sequences of ureB, hspA, and ureB-hspA were amplified by PCR from the vaccine SH02, cloned into an expression vector pGEX-6P-1(+) plasmid (GE Healthcare, Pittsburgh, USA), and placed between *BamH*I and *Not*I restriction sites (UreB-F: 5′-CGCGGATCCAAAAAGATTAGCAGAAAAG-3′, UreB-R: 5′-AAATATGCGGCCGCCTAGAAAATGCTAAAGAG-3′; HspA-F: 5′-CGCGGATCCAAGTTTCAACCATTAG-3′, HspA-R: 5′-AAATATGCGGCCGCTTAGTGTTTTTTGTGATC-3′; UreB-HspA-F: 5′- CGCGGATCCAAAAAGATTAGCAGAAAAG-3′, UreB-HspA-R: 5′- AAATATGCGGCCGCTTAGTGTTTTTTGTGATC-3′). The recombinant plasmid was transformed into *Escherichia coli* (*E. coli*) BL21 (DE3) cells (Cwbio, Suzhou, China), and protein expression was subsequently induced with 1 mM IPTG at 20°C before being harvested after 18 hours by centrifugation. The bacterial pellets were resuspended in PBS and sonicated. Any insoluble cellular components were removed by centrifugation, and the rGST-UreB, rGST-HspA, and rGST-UreB-HspA fusion proteins were purified by Price Glutathione Superflow Agarose (Thermo, Rockford, USA) according to the manufacturer’s protocol. Moreover, the GST tag was excised with PreScission Protease (Beyotime, Nanjing, China) and removed with GST-tag Purification resin (Beyotime, Nanjing, China). Finally, the purity and concentration of the recombinant protein rUreB, rHspA, and rUreB-HspA were determined *via* SDS-PAGE, Western Blot, and BCA assay.

### SDS-PAGE and Western Blot Analysis

The UreB-HspA expressed by the vaccine strain SH02 and the rUreB-HspA expressed and purified from *E. coli* were verified by Western Blot analysis. The vector strain without antigen expression, SH02, purified rUreB, rHspA, and rUreB-HspA were denatured and separated by 15% SDS-PAGE, transferred onto a polyvinylidene fluoride (PVDF) membrane (Merck Millipore, Mannheim, Germany), and then blocked with PBST containing 5% skimmed milk at 37°C for one hour to prevent non-specific protein binding. The membranes were incubated with appropriate dilution of UreB monoclonal antibodies (21) or immunized mice sera at 37°C for one hour, washed with PBST three times, and then incubated with HRP-conjugated rabbit anti-mouse IgG (1:5000 dilution, Abcam, Cambridge, UK) at 37°C for one hour. The HRP Western Blot Analysis Kit (Easybio, Beijing, China) was used to detect the binding reaction.

### Immunization and Sample Collection

Three groups of six-to-eight-week-old, female, specific-pathogen-free (SPF) BALB/c mice (N = 10/groups) were immunized three times on days 0, 14, and 42. Group A received oral SH02 for all three immunizations, and Group B received oral SH02 for the first two immunizations and a subcutaneous injection of 50 µg 0.05 mL rUreB-HspA + 0.05 mL AddaVax for the third immunization. The control group received oral PBS for all three immunizations. After 20 min of neutralization of gastric acid with 0.5 ml 3% NaHCO_3_, all mice were orally inoculated with 2.5 × 10^9^ CFU of SH02 with a volume of 0.2 mL, 0.2 mL PBS, or injected subcutaneously with rUreB-HspA + AddaVax. On day 56, all mice were challenged with 1.3 × 10^8^ CFU of *H. pylori* strains SS1 with a volume of 0.2 mL. The concentration of SS1 was confirmed by plating 10-fold serial dilutions on campylobacter agar base plates (CDRC, Shanghai, China), and cultured as above for three days.

Before they were challenged, serum was collected for the detection of serum-specific antibody levels, and fresh fecal pellets were collected for the determination of specific secretory IgA (sIgA) levels. The fecal samples were weighed and then suspended in PBS at a ratio of 0.5 mL/100 mg fecal pellets. Moreover, the sample was homogenized by vortexing at room temperature for five min, then being centrifuged at 8000 × g for 10 min, with the supernatant transferred to a new tube containing 1% protease inhibitor cocktail (Bimake, Shanghai, China). Eight weeks following the challenge, all mice were sacrificed and their stomachs were taken out aseptically for determination of *H. pylori* load and pathological histology analysis. Spleens were simultaneously removed for performing lymphocyte proliferation assays and flow cytometry.

All animals were purchased from the Vital River Laboratory (Beijing, China) and bred under SPF conditions. Animal experiments were confirmed by the Animal Ethics Committees and performed under the regulations of the Animal Care and Use Committee guidelines from the Academy of Military Medical Sciences.

### Specific Antibody Detection by ELISA

Antigen-specific IgG, IgG1, IgG2a, and IgG2b levels in the sera and sIgA levels in the fecal supernatant were determined by ELISA. The ELISA plates were coated with rUreB-HspA (100 µL, 5 µg/mL) diluted in 0.05 M carbonate coating buffer. The plates were incubated overnight at 4°C, washed three times with PBST, and then blocked with a blocking buffer (PBST containing 5% skimmed milk) for one hour at 37°C. The blocking solution was discarded, and serially diluted serum samples and fecal supernatant were added to the plates. After incubating at 37°C for one hour, the plates were washed again and horseradish peroxidase (HRP) conjugated anti-mouse sIgA, IgG, IgG2b, IgG1 or IgG2a (Abcam, Cambridge, UK) antibody (100 μL/well) was added. The plate was incubated again at 37°C for one hour and washed, and 3,3,5,5-tetramethylbenzene-biphenyl (TMB) substrate (Solarbio, Beijing, China) was subsequently added at 100 μL/well. After the reaction was carried out in the dark for 15–20 min, 1 M H_2_SO_4_ was added to stop the reaction, and the A450 of each well was measured in SpectraMax i3x (Molecular Devices, San Jose, USA). All samples were analyzed in duplicate, and the means of the replicates were reported in the results.

### *H. pylori* Culture Count

Stomachs were taken out aseptically, cut along the large bend from the pylorus to the cardia, and placed flat in a sterile petri dish with the mucosa facing upward. The gastric mucosal surface was subsequently flushed with sterile saline through a syringe. Half of the stomach was weighed and transferred to a homogenization tube containing brucella broth at a ratio of 1: 10 and then homogenized with a tissue homogenizer. The colonization level of *H. pylori* was determined by applying a 10-fold dilution of the gastric homogenate to a campylobacter agar plate (CDRC, Shanghai, China) containing 10 µg/mL amphotericin B, 10 µg/mL vancomycin, 20 U/mL bacitracin, 10 µg/mL polymyxin B, and 5 µg/mL trimethoprim, and culturing for seven days as described above. CFUs were enumerated and bacteriology data were expressed as CFU/g stomach.

### Histopathology

The other half of the stomach tissue sample was fixed in 10% neutral buffered formalin, then processed and embedded in paraffin. The sections were cut 4 mm thick and stained with hematoxylin-eosin, and the severity of gastritis (infiltration degree of lymphocytes) was determined by an experimental pathologist who did not know the distribution of the experimental group. The extent of gastritis was graded as follows: 0, none; 1, a small amount of lymphocytes infiltrated the lamina propria; 2, a moderate amount of lymphocytes infiltrated the lamina propria; 3, lymphocytes densely infiltrate the lamina propria, and some enter the submucosa; 4, dense, diffuse lymphocytic infiltration of the lamina propria and submucosa.

### Lymphocyte Proliferation Assay

Spleens were aseptically removed and pressed through a fine nylon mesh using syringe plunges to prepare single cell suspensions. Cells were seeded (4 × 10^5^ cells per well) in RPMI 1,640 medium (Giboc, Grand Island, USA) containing 10% FBS and 100 U/mL Penicillin-Streptomycin in the presence of 10 μg/mL rUreB-HspA (Endotoxin < 100 EU/mg) for 72 hours at 37°C and 5% CO_2_. Under the same conditions, the cells without rUreB-HspA were regarded as non-antigen-stimulated cells. The cells were pulsed with 20 μL Cell Counting Kit-8 (CCK8) solution (Dojindo, Kumamoto Ken, Japan) per well for the last four hours of culturing, and the absorbance at 450 nm (A450) was measured in SpectraMax i3x (Molecular Devices, San Jose, USA). The results were expressed as stimulation indexes (SI): SI = OD value of stimulated cultures/OD value of non-stimulated cultures.

### Flow Cytometer Analysis

Single-cell suspensions of 4 × 10^6^ spleen cells were plated in 12-well plates, stimulated with 2 µg CD28 and 40 µg rUreB-HspA (Endotoxin < 100 EU/mg), supplemented with 100 IU/mL Penicillin-Streptomycin and 10% FBS in 2 mL RPMI 1,640 medium, and incubated for 24 hours at 37°C in 5% CO_2_. In the last six hours, protein transport inhibitors Brefeldin A (BD, New Jersey, USA) 2 µL/mL and Monensin (BD, New Jersey, USA) 1.4 µL/mL were added to the cells. After centrifugation to remove the supernatant, 2 μL Fc receptor blocker (Biolegend, California, USA) was added to the cells, which were incubated for 10 min at room temperature, with 5 μL premixed True-Stain Monocyte Blocker (Biolegend, California, USA) and 2 μL CD4 (Biolegend, California, USA) subsequently added before being incubated again at room temperature in the dark for 25 min. After washing the cells with PBS, 1 mL diluted Zombie Aqua solution (Biolegend, California, USA) was added, and the cells were incubated for 10 min and centrifuged. The cells were then fixed, permeabilized, and washed with 2 mL BD Perm/Wash™ Buffer using the Fixation/Permeabilization Kit (BD, New Jersey, USA) according to the manufacturer’s instructions. Following each centrifugation, the cell cluster was ejected. Next, pre-mixed Brilliant Stain Buffer Plus (BD, 10 μL), True-Stain Monocyte Blocker (5 μL), CD3 (Biolegend, California, USA), CD154 (Biolegend, California, USA), IL-17A (Biolegend, California, USA), and IFN-γ (Biolegend, California, USA) (2 μL for each antibody) were added to the cells, thoroughly mixed, and the cells were incubated for 30 min at room temperature in the dark. Finally, the cells were washed with BD Perm/Wash™ Buffer and PBS, resuspended in 0.2 mL PBS, and the number of various types of cells was measured on the Cytek-Aurora flow cytometer.

### Statistical Analysis

All data were represented as mean ± standard deviation (SD). D’Agostino-Pearson normality test in Normality and Log normality were used to test whether the data conformed to a normal distribution before statistical analysis. When the experimental data conformed to the normal distribution, one-way ANOVA followed by Bonferroni multiple pairwise comparison tests or Brown-Forsythe and Welch ANOVA followed by Tamhane2 were used to analyze the differences between the groups. The non-parameter Mann-Whitney test was used to analyze non-normally distributed data. All statistical analyses were performed using GraphPad Prism version 8.0.2, and the difference was considered significant at *p* < 0.05.

## Results

### Preparation of Candidate Vaccine SH02 and Purification of rUreB-HspA for Inoculating Mice

**Figure 1:**
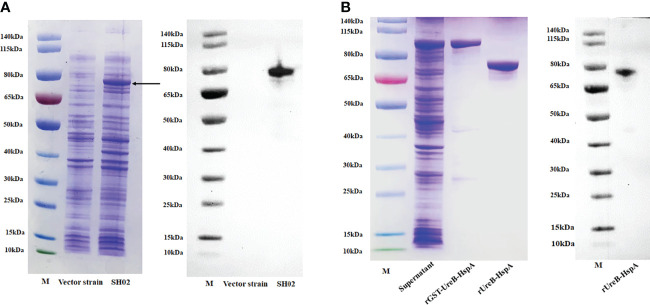
Expression, purification, and validation of proteins. **(A)** UreB-HspA was expressed in SH02 strain (left) and validated with UreB monoclonal antibody (right). The bond of UreB-HspA was marked by black arrow. Vector strain served as negative control. M: Marker; Lane 1: Vector strain; Lane 2: Candidate vaccine strain SH02. **(B)** Expression and purification of rGST-UreB-HspA (left), verified with UreB monoclonal antibody (right) after GST tag removal. M: Marker; Lane 1: Supernatant of ultrasonic broken material of *E*. *coli* BL21 (pGEX-6P-UreB-HspA); Lane 2: rGST-UreB-HspA; Lane 3: rUreB-HspA.

Prior to each immunization of mice, we took a new cryopreserved candidate vaccine strain SH02 to induce expression and confirmed the expression of the fusion antigen UreB-HspA by SDS-PAGE and Western Blot (**Figure 1A**). The recombinant protein rUreB-HspA was correctly expressed in recombinant clone *E. coli* BL21 (pGEX-6P-UreB-HspA). After purification and removal of GST tag, rUreB-HspA was verified by SDS-PAGE and Western Blot (**Figure 1B**).

### Immunization and Detection of Antigen-Specific Antibody

**Figure 2:**
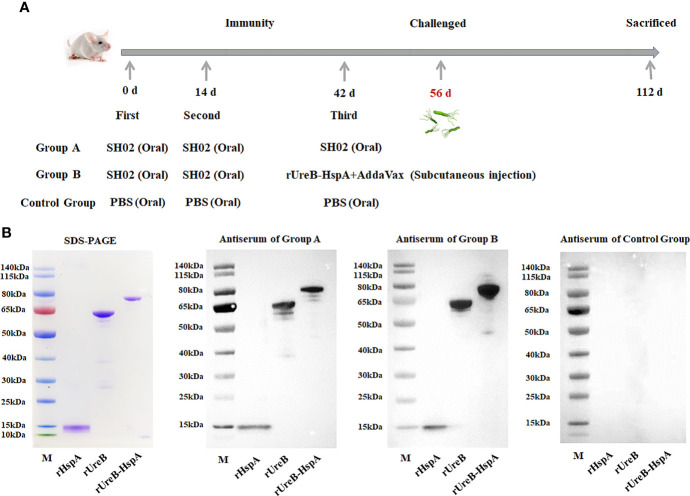
Immunization schedule and antigen-specific antibody detection. **(A)** Group A mice received oral administration of SH02 on days 0, 14, and 42; Group B mice received oral administration of SH02 on days 0 and 14, and subcutaneous injection of rUreB-HspA + adjuvant on day 42; oral administration of PBS three times served as a control group. Two weeks following the final vaccination, the mice were infected with live *H*. *pylori*, and eight weeks later, they were sacrificed and samples were collected. **(B)** The antigen-specific antibodies in the sera of immunized mice in the three groups before challenge were detected by Western Blot. M: Marker; Lane 1: rHspA; Lane 2: rUreB; Lane 3: rUreB-HspA.

Three groups of mice were immunized and challenged as described in the method, and the detailed immunization schedule was shown in **Figure 2A**. Two weeks following the final immunization and before the challenge, we first evaluated the antigen-specific antibody produced by immunized mice by Western Blot (**Figure 2B**).

**Figure 3:**
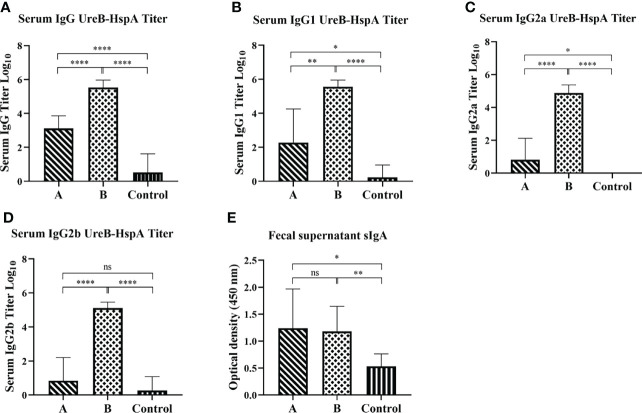
Mucosal and humoral immune responses induced by immunization. Two weeks following the final immunization, serum and feces of the mice were collected before the challenge and the antigen-specific IgG **(A)**, IgG1 **(B)**, IgG2a **(C)**, IgG2b **(D)**, and sIgA **(E)** levels were measured by ELISA. The data were presented as mean ± SD, and differences between groups were tested using one-way ANOVA followed by Bonferroni’s multiple-pair comparison test. *p < 0.05, **p < 0.01, ****p < 0.0001.

Next, we detected the levels of mucosal and humoral immune responses induced by immunization and the IgG antibody subtypes by ELISA. As expected, compared with the control group, the non-subcutaneous booster injection group (Group A) and the subcutaneous booster injection group (Group B) both produced UreB-HspA-specific IgG antibodies (*p* < 0.0001). Moreover, the serum antigen-specific IgG titer of Group B was significantly increased, which was markedly different from that of Group A (*p* < 0.0001). (**Figure 3A**) To further characterize the type of immune responses induced by the vaccine candidate, sera were analyzed for the antigen-specific IgG subtypes (IgG1, IgG2a, and IgG2b). As shown in **Figures 3B–D**, oral candidate vaccine SH02 (Group A) can cause IgG1 and IgG2a responses, and the subcutaneous injection of rUreB-HspA to boost immunity in Group B can cause IgG1, IgG2a, and IgG2b responses. On the other hand, we tested the antigen-specific mucosal sIgA antibody levels in the feces of mice two weeks following immunization. The results indicated that compared with the control group, groups A and B both produced a significant mucosal immune response (*p* < 0.01), but there was no difference between group A and group B (*p* > 0.05) (**Figure 3E**).

### Prophylactic Protection Against *H. pylori* Infection

**Figure 4:**
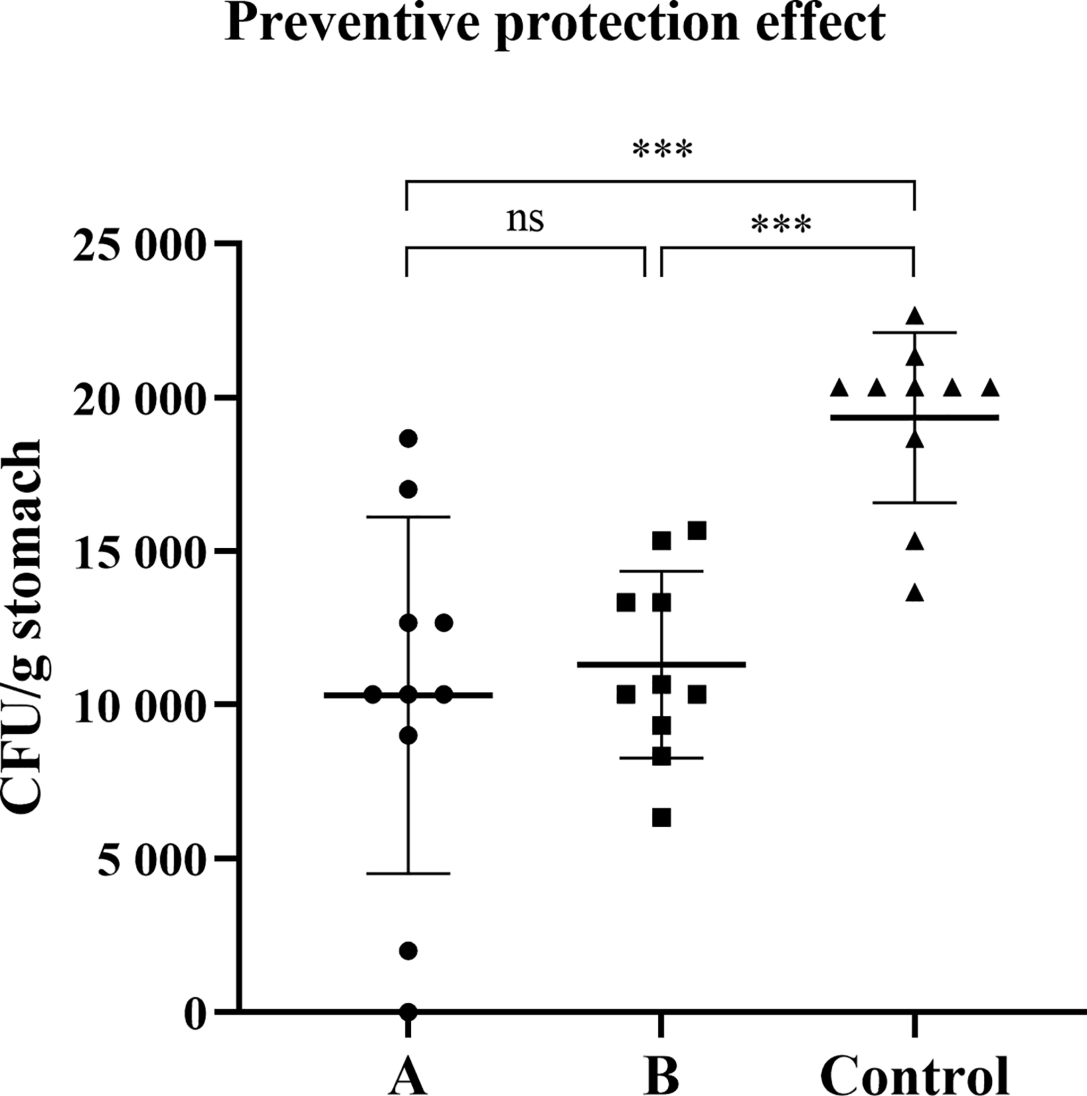
Preventive immunization can induce protection against *H. pylori* challenge. Two weeks after the final immunization, the mice were infected with live *H. pylori* SS1 bacteria. After eight weeks of the challenge, the colonization of *H. pylori* was confirmed by quantitative culture. The data were presented in the form of a scatter plot. Each symbol represents a mouse, and the horizontal line represented the mean ± SD, and differences between groups were tested using one-way ANOVA followed by Bonferroni’s multiple-pair comparison test. ***p < 0.001. ns *p*>0.05.

Since oral vaccine candidate SH02, both with or without subcutaneous injection of rUreB-HspA, demonstrated strong immunogenicity, we investigated whether immunization can prevent *H. pylori* infection. Two weeks after the final immunization, the mice were challenged with live *H. pylori*, and eight weeks following the challenge, the amount of bacterial colonization in the stomach of the mice was evaluated. Compared with the control group, the amount of *H. pylori* colonization in groups A and B was significantly reduced (*p* < 0.001). However, there was no significant difference between groups A and B (*p* > 0.05) (**Figure 4**).

**Figure 5:**
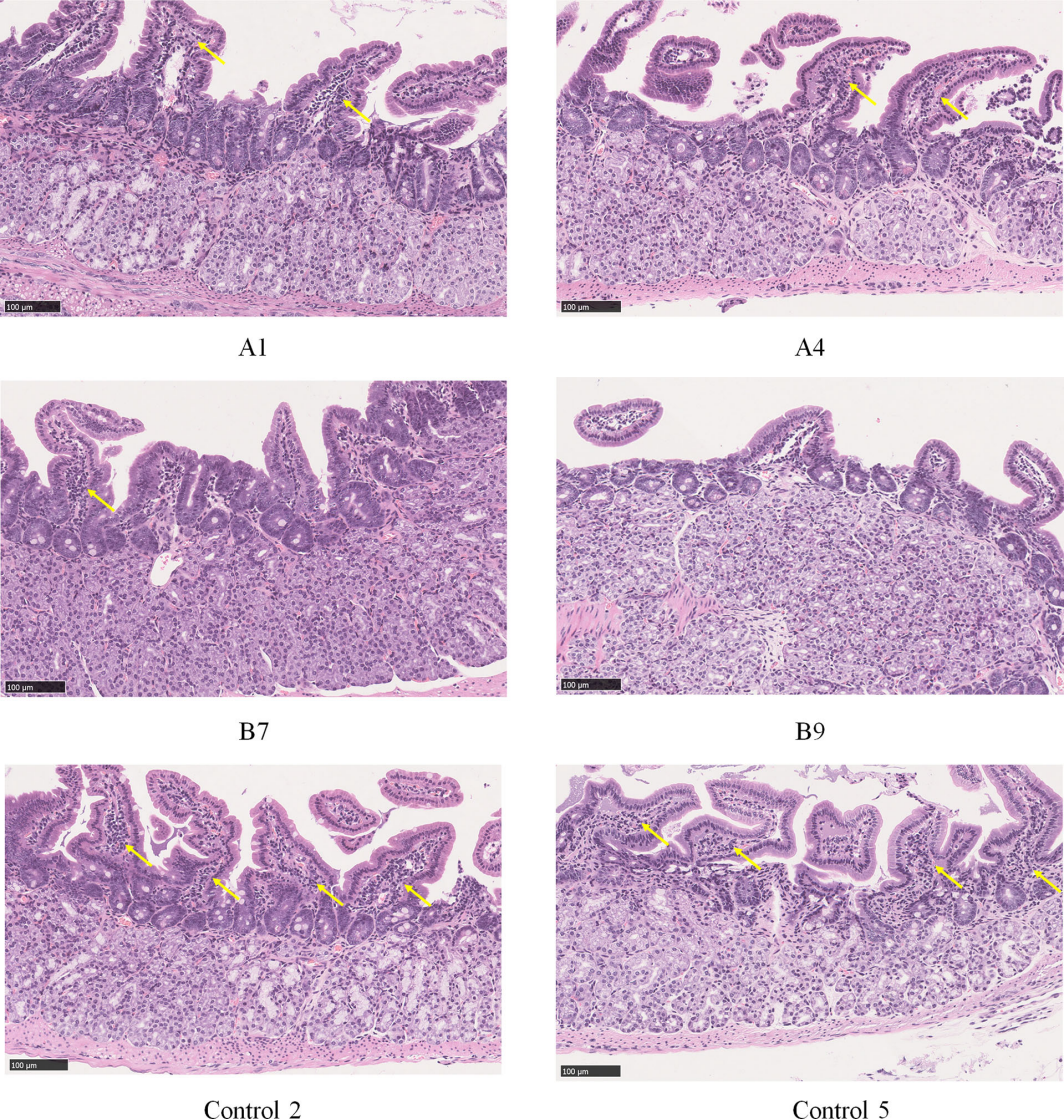
Histopathological pictures of gastric antrum mucosa in some experimental mice (HE staining). Group A: a small amount of lymphocyte infiltration and mild inflammation in the gastric antrum mucosa of mice A1 and A4; Group B: a small amount of lymphocyte infiltration and mild inflammation in the gastric antrum mucosa of mouse B7, and no obvious abnormality in mouse B9; Control group: moderate lymphocyte infiltration and moderate inflammation were seen in the lamina propria of gastric antrum mucosa of mice 2 and 5 in the control group. Locations of lymphocyte aggregation were indicated by yellow arrows.

The histopathological examination of the stomach tissues of the immunized mice sacrificed eight weeks after the *H. pylori* challenge indicated that the tissue structure of the stomach was not abnormal in the three experimental animal groups. The lymphocytes were located in the lamina propria of the gastric antrum. The control group (9/10) exhibited a wider range of inflammation with the most severe lesions, and Group A (6/10) exhibited milder inflammation than the control group. The inflammation in Group B was the mildest, with only four out of 10 mice exhibiting mild local chronic inflammation. The inflammation scores of all mice were shown in **Table 1**, and the histopathological pictures of gastric antrum mucosa in some experimental mice were shown in **Figure 5**.

**Table 1:** Stomach inflammation score.

Mouse number	Group A	Group B	Control group
1	1	1	2
2	0	0	2
3	1	1	1
4	1	1	1
5	0	0	2
6	0	0	0
7	1	0	2
8	0	1	1
9	1	0	1
10	1	0	1

Inflammation scoring criteria: 0, none; 1, a small amount of lymphocytes infiltrated the lamina propria; 2, a moderate amount of lymphocytes infiltrated the lamina propria; 3, lymphocytes densely infiltrate the lamina propria, and some enter the submucosa; 4, dense, diffuse lymphocytic infiltration of the lamina propria and submucosa.

### Lymphocyte Proliferation Test

**Figure 6:**
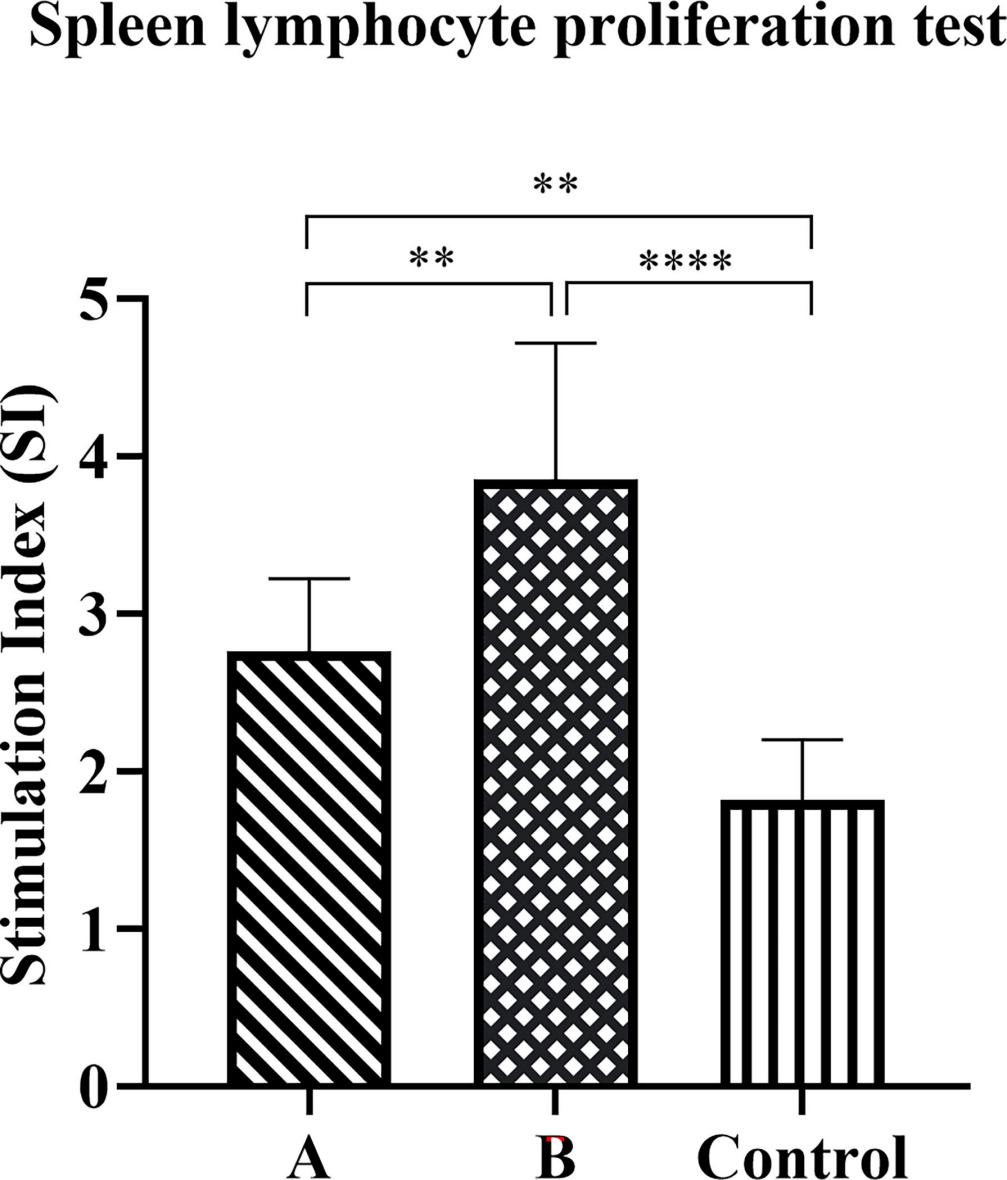
Antigen-specific lymphocyte proliferation response. The spleen was collected eight weeks after the challenge to prepare a single-cell suspension. Cells were cultured in the presence of candidate antigens or medium only, and proliferation was measured using the CCK8 method. The data were presented as mean ± SD, and differences between groups were tested using one-way ANOVA followed by Bonferroni’s multiple-pair comparison test. **p < 0.01, ****p < 0.0001.

We evaluated the proliferative response of splenic lymphocytes in each group of mice after the *H. pylori* challenge. Compared with control group mice, the proliferation of lymphocytes in groups A and B increased significantly eight weeks after the challenge (**Figure 6**). Moreover, the proliferation of lymphocytes in Group B was significantly higher than that of Group A (*p* < 0.01).

### Antigen-Specific T Cell Response

**Figure 7:**
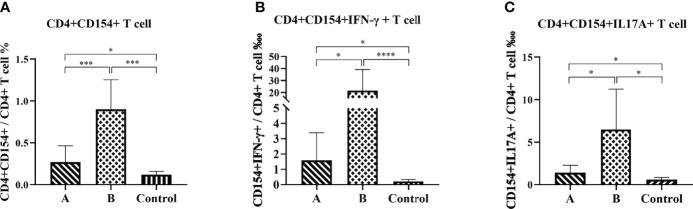
Antigen-specific CD4+ T cell immune response. Eight weeks after the *H. pylori* challenge, the spleens were collected to prepare a single cell suspension. After staining with intracellular cytokines, flow cytometry was used to determine the proportion of CD4+CD154+ T cells **(A)**, CD4+CD154+IFN-γ+ T cells **(B)**, and CD4+CD154+IL17A+ T cells **(C)** in total CD4+ T cells. The data were presented as mean ± SD. Brown-Forsythe and Welch ANOVA followed by Tamhane2 were used to analyze the data in panels **(A–C)** The nonparametric Mann-Whitney test was used to analyze the data in **(B)** *p < 0.05, ***p < 0.001, ****p < 0.0001.

Flow cytometry analysis was performed to compare antigen-specific spleen T lymphocyte responses in the immunized and control mice at eight weeks after the *H. pylori* challenge. In this study, we used the rUreB-HspA to stimulate splenic lymphocytes, and analyzed the level of CD4+CD154+ T cells and CD4+CD154+ T cells that secrete IFN-γ or IL-17A, which were antigen-specific T lymphocytes. The results demonstrated that the frequency of UreB-HspA-specific CD4+CD154+ T cells, CD4+CD154+IFN-γ+ T cells, and CD4+CD154+IL17A+ T cells in the spleens of mice in groups A and B increased significantly, with Group B significantly higher than Group A (**Figure 7**).

## Discussion

Natural *H. pylori* infection does not trigger a protective immune response that can lead to the eradication of the infection; therefore, chronic infection remains in most infected people. Although most infections are asymptomatic, about 10–20% of infected individuals will develop peptic ulcer and 1–2% will develop gastric cancer and mucosa-associated lymphoid tissue (MALT) lymphoma (22). At present, the exact protective immune mechanisms against *H. pylori* are still not very clear (23). The development of preventive and therapeutic *H. pylori* vaccines has obtained encouraging protection results in animal models (24–26), with some vaccines entering clinical studies (27–29). However, most candidate vaccines are still in the early stages of development, and there are still difficulties and challenges in the development of safe and effective *H. pylori* vaccines.

Humans are the only host of *H. pylori*, and so far there is no perfect animal model for research. The mouse-adapted *H. pylori* Sydney strain SS1 or SS2000 challenge mouse test to evaluate vaccine protection is currently the most widely used animal model. *H. pylori* is mainly colonized on the surface of epithelial cells of gastric mucosa, so induction of mucosal immune responses against *H. pylori* is an effective way to develop vaccines. However, the microenvironment of gastrointestinal mucosal immunity is complex, and antigen molecules are easily degraded. Mucosal immunization with live attenuated bacteria carrier vaccine can avoid the degradation of antigen in gastrointestinal tract, effectively transfer antigen to mucosal immune cells, and have adjuvant effect at the same time. The efficiency of prime-boost regimes to enhance protective immunity has been increasingly recognized and different successful prime-boost combinations have been reported for a number of vaccine candidates (30). Traditionally the same vaccines are given multiple times as homologous boosts. In many cases, heterologous prime-boost with different types of vaccines containing the same antigens can be more immunogenic than homologous prime-boost. In a previous study, we developed a recombinant live vector vaccine SH02 against *H. pylori* using attenuated *Shigella flexneri* 2aT32 as antigen delivery vector. In this research, we designed two different prime-boost strategies: two oral immunization as the basic immunization, followed by homologous boost or heterologous boost. We detected the immunogenicity of orally immunized mice with the candidate vaccine strain SH02, and evaluated whether it was possible to further enhance the level of mucosal, humoral and cellular immune response and the effect of immune prevention by employing the heterologous prime-boost strategy with AddaVax adjuvants deliver rUreB-HspA antigens parenterally. AddaVax is similar to MF59, an oil-in-water emulsion adjuvant composed of squalene, Tween 80, and Span 85, which has been approved in Europe for human influenza vaccines (31).

After three immunizations, we evaluated the colonization level of *H. pylori* in the mouse stomach and gastric histopathology changes eight weeks following immunization and *H. pylori* challenge. Compared with the control group, the colonization levels of *H. pylori* in the stomach of mice in groups A and B were significantly reduced (*p* < 0.001). Histopathological analysis demonstrated that extensive lymphocytic infiltration and chronic inflammation occurred in the gastric antrum of mice in the control group, and the degree of inflammation and lymphocyte infiltration in Group A was less than that in the control group. The degree of inflammation in Group B was the least, with only a few animals exhibiting mild localized chronic inflammation and lymphocyte infiltration. These results indicated that the candidate vaccine strain SH02 possesses the effect of inducing protective immunity against *H. pylori*. Although the levels of bacterial colonization in the stomach of Group B was not significantly different from that of Group A, following subcutaneous injection of rUreB-HspA for immune enhancement, the degree of gastric inflammation and lymphocyte infiltration were significantly reduced, indicating that subcutaneous injection of candidate antigens can reduce the inflammatory response caused by *H. pylori* infection and enhance the protective effect of SH02.

To explore the potential mechanism of immune protection of candidate vaccines, we tested antigen-specific serum IgG, fecal sIgA antibodies and cellular immunity following immunization. Previous research indicated that anti-*H. pylori*-specific sIgA from milk can protect infants from *H. pylori* infection for a long time (32) or delay the acquisition of *H. pylori* infection (33), indicating that pre-existing *H. pylori* specific sIgA may help prevent infection. In our study, the oral candidate vaccine SH02 could induce mice to produce antigen-specific IgG and sIgA antibodies. When boosted with rUreB-HspA, the serum IgG titer increased significantly, but fecal sIgA levels were not. These results indicated that the booster of injection immunization can enhance systemic immune responses, but not mucosal immune responses. In recent years, some studies have shown that the prevention of *H. pylori* infection is mediated by mixed Th cell response (24, 34, 35). B lymphocytes produce specific antibodies related to Th1 response such as IgG2a and sIgA, and antibodies related to Th2 response such as IgG1. The balanced Th1/Th2 response is essential to clear *H. pylori* infection (36). In this study, we analyzed the antibody subtypes against UreB-HspA in immunized mice and determined that the immunization produced both antigen-specific IgG1 and IgG2a antibodies, which indicated that the immune response triggered by vaccination is a mixed Th1/Th2 type.

The role of CD4+ T cells in preventing *H. pylori* infection has been widely accepted. Assessing the expression of CD40 ligand (CD154) on the surface of activated CD4+ T cells can be used to identify a complete set of antigen-specific cells, regardless of their cytokine expression profile (37). In our study, the splenic lymphocytes of mice were stimulated by rUreB-HspA and labeled CD4 and CD154. We found that both groups A and B produced significantly increased antigen-specific CD4+ T cells compared with the control group, and the proportion of CD4+CD154+ T cells in Group B was significantly higher than that in Group A (*p* < 0.001). Numerous studies have confirmed that IFN-γ and IL-17A, as well as T cells that secrete these cytokines, play an important role in protective immunity against *H. pylori* (25, 26, 38). Therefore, we screened CD4+CD154+ T cells that secrete IFN-γ and IL-17A. With similar results, the proportion of CD4+CD154+IFN-γ+ T cells and CD4+CD154+IL17A+ T cells in groups A and B increased significantly, with Group B being higher than Group A. Moreover, lymphocyte proliferation experiments also confirmed that immunization induced antigen-specific lymphocyte responses, with a stronger response observed in Group B than Group A. Stronger T lymphocyte immune response and humoral immune response may be the reason for the reduction of gastric inflammation in Group B, although there was no difference in the levels of bacterial colonization between the two groups. Moreover, this phenomenon and potential mechanism need to be further explored.

## Conclusion

Our work confirmed that the candidate vaccine SH02 demonstrates relatively strong immunogenicity. Following oral immunization, the levels of *H. pylori* colonization and gastric inflammation in mice were significantly reduced, and the parenteral rUreB-HspA booster further enhanced humoral and cellular immunity against *H. pylori* and reduced gastric inflammation. The potential immune protection against *H. pylori* infection may require the combined action of mixed cellular immunity and IgG humoral immunity, as well as sIgA mucosal immunity response.

## Data Availability Statement

The original contributions presented in the study are included in the article/supplementary material. Further inquiries can be directed to the corresponding authors.

## Ethics Statement

The animal study was reviewed and approved by Animal Ethics Committees of the Academy of Military Medical Sciences.

## Author Contributions

Conceptualization, CL and YW; methodology and validation, XZ, QG, and HT; formal analysis and data curation, XZ, CL, YW, and SS; writing—original draft preparation, XZ; writing—review and editing, CL and YW. All authors have read and agreed to the published version of the manuscript.

## Funding

This research was funded by National Science and Technology Major Project for Prevention and Treatment of Infectious Diseases, grant number 2018ZX10101003-005-005.

## Conflict of Interest

The authors declare that the research was conducted in the absence of any commercial or financial relationships that could be construed as a potential conflict of interest.

## Publisher’s Note

All claims expressed in this article are solely those of the authors and do not necessarily represent those of their affiliated organizations, or those of the publisher, the editors and the reviewers. Any product that may be evaluated in this article, or claim that may be made by its manufacturer, is not guaranteed or endorsed by the publisher.
